# Vessel Wall Inflammatory Activity as Determined by F-18 Fluorodeoxyglucose PET in Large Vessel Vasculitis Is Attenuated by Immunomodulatory Drugs

**DOI:** 10.3390/diagnostics11071132

**Published:** 2021-06-22

**Authors:** Romilda Sherzay, Torsten Witte, Thorsten Derlin, Marius Hoepfner, Frank M. Bengel

**Affiliations:** 1 Department of Nuclear Medicine, Hannover Medical School, 30625 Hannover, Germany; sherzay.romilda@mh-hannover.de (R.S.); derlin.thorsten@mh-hannover.de (T.D.); 2 Department of Rheumatology and Immunology, Hannover Medical School, 30625 Hannover, Germany; Witte.Torsten@mh-hannover.de (T.W.); Hoepfner.Marius@mh-hannover.de (M.H.)

**Keywords:** large vessel vasculitis, vasculature, F-18 FDG, inflammation, immunomodulation

## Abstract

F-18 fluorodeoxyglucose (F-18 FDG) PET/CT plays an increasing role in the diagnostic workup of large vessel vasculitis (LVV); however, information on the relationship between immunosuppressive drugs and vessel wall uptake is limited. In 94 patients with a confirmed diagnosis of LVV, the vessel wall-to-liver ratio (VLR) was assessed in eight vessel segments. Patients were grouped according to intake of immunomodulatory drugs (Group 1, prednisone; Group 2, prednisone + methotrexate; and Group 3, prednisone + others) and compared to treatment-naïve individuals. A total of 54/94 (57.4%) were treated with immunomodulatory drugs (Group 1, 29/49 (53.7%); Group 2, 9/54 (16.7%); Group 3, 11/54 (20.4%); and Group 4, 5/54 (9.3%)), whereas the remainder received no therapy (40/94 (42.6%)). The mean VLR of the arterial segments correlated significantly with the hematopoietic organs (*r* ≥ 0.22, *p* ≤ 0.05), c-reactive protein (*r* ≥ 0.25, *p* ≤ 0.05), and prednisone dosage (*r* ≥ −0.4, *p* ≤ 0.05). Relative to treatment-naïve patients, a significantly lower VLR was recorded in 5/8 (62.5%) of the investigated vessel segments in Group 1 (*p* ≤ 0.02), in 6/8 of the vessel segments in Group 2 (75.0%, *p* ≤ 0.006), and in 7/8 of the segments in Group 3 (87.5%, *p* ≤ 0.05). In LVV, the F-18 FDG uptake in vessel wall as a marker of inflammatory activity was attenuated by immunomodulatory drugs, which provides a foundation for future serial monitoring of treatment efficacy.

## 1. Introduction

In patients with suspected large vessel vasculitis (LVV), rapid and early comprehensive imaging testing is recommended to complement the clinical criteria and to establish diagnosis [1]. Serving as a whole-body non-invasive read-out potentially assessing the entire vasculature, F-18 fluorodeoxyglucose (F-18 FDG) PET/CT is strongly endorsed by the current guidelines for initial diagnostic work-up [2]. The induction treatment, however, should not be delayed by imaging. Accordingly, at the time of the scan, patients may already be under immunosuppressive therapy [3], including glucocorticoids (GC), initiated for remission or methotrexate at high risk for relapse [4].

The majority of these patients, however, are not able to substantially lower GC doses, and thus the early administration of GC-sparing agents would be desirable [4]. In this regard, sustained remissions in combination with a prednisone taper were achieved under tumor-necrosis factor-inhibitors [5], mycophenolate mofetil [6], leflunomide [7], or tocilizumab [8]. Thus far only the effect of GC on the vessel wall uptake in LVV has been reported, which showed a decrease F-18 FDG uptake [9].

In clinical practice, several immunomodulatory drugs are prescribed, particularly if the symptoms persist with GC [10]. Thus, given the broad range of immunosuppressive drugs used for LVV treatment, we aimed to evaluate the impact of immunosuppressive therapy on vessel wall uptake as measured by F-18 FDG uptake at the time of PET/CT. Demonstrating a relationship between immunomodulating drugs and the F-18 FDG uptake in arterial segments may, therefore, lay the foundation for future PET-guided therapy in LVV.

## 2. Materials and Methods

### 2.1. Study Cohort

From January 2014 to December 2018, 94 patients with a clinical diagnosis of LVV, who had either persistent symptoms (e.g., headache) or demonstrated increased c-reactive protein (CRP) at routine laboratory work-up, were referred for F-18 FDG PET/CT. Any other cause for CRP elevation was ruled out prior to the imaging test. The patient characteristics are displayed in Table 1.

For patients with ongoing immunomodulatory therapy, data on steroid and other immunosuppressive therapies were obtained at the time of PET/CT. The patients were then grouped according to the type of medication as follows: Group 1 taking prednisone daily; Group 2 with daily prednisone and methotrexate intake (once per week); and Group 3 receiving daily prednisone plus other immunomodulatory drugs, including daily intake of mycophenolate mofetil, azathioprine, leflunomide, and rituximab every second week).

**Figure 1:**
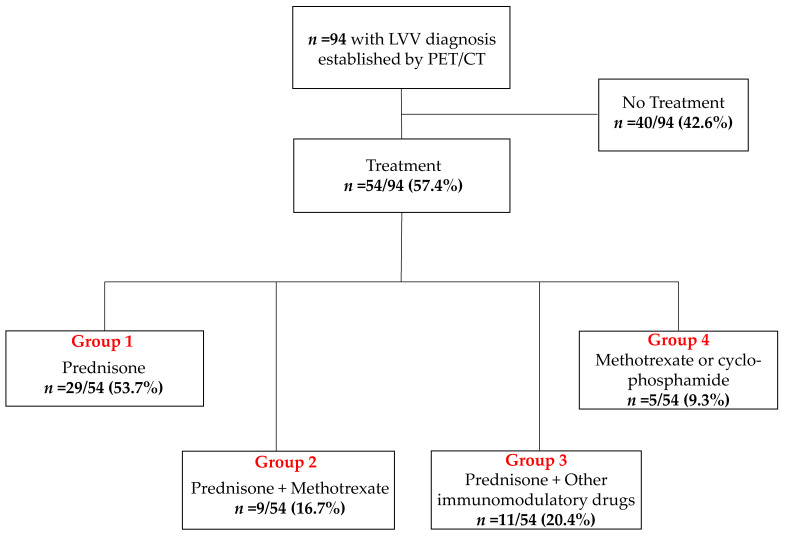
Overview of the enrolled subjects. Other immunomodulatory drugs in Group 3 included mycophenolate mofetil, azathioprine, rituximab, and leflunomide. Patients allocated to Group 4 were excluded from further analysis due to the low number of subjects. LVV = large vessel vasculitis.

In Group 4, none of the patients received GC, instead they received methotrexate or cyclophosphamide alone, and this group was excluded from further analyses due to the low number of subjects assigned to this group (Figure 1). The CRP levels and the daily GC doses at the time of PET/CT were recorded. The treatment duration could not be established in all subjects given the retrospective nature of the present study.

### 2.2. PET/CT Image Analysis

Integrated F-18 FDG PET/CT was performed in all patients using a dedicated PET/CT system (Biograph mCT 128 Flow; Siemens, Erlangen, Germany), equipped with an extended field-of-view PET component, a 128-slice spiral CT component, and a magnetically driven table optimized for continuous scanning. Before image acquisition, the patients fasted for at least 6 h, and their blood glucose levels were less than 160 mg/dL. F-18 FDG (261 ± 52.5 MBq) was injected intravenously in all patients (injected 60 min prior to acquisition).

Imaging started with a low-dose nonenhanced helical CT (120 kV, mA modulated, pitch of 1.4, and reconstructed axial slice thickness of 5.0 mm) for attenuation correction. Whole-body PET images were subsequently acquired using continuous bed motion at a speed of 1.7 mm/s for the head, 1.3 mm/s for the chest and abdomen, and 2.5 mm/s for the legs at 1 h after injection. All studies were reconstructed using Ultra HD, an iterative algorithm combined with time-of-flight and point-spread function information (Siemens Healthcare, Erlangen, Germany; 2 iterations, 21 subsets; matrix, 200; zoom, 1.0; and Gaussian filter, 5.0).

The PET images were analyzed using a dedicated workstation (Syngo.Via; V10B; Siemens Healthcare, Erlangen, Germany), allowing a simultaneous and fused review of the PET and CT data. PET, CT, and hybrid PET/CT imaging overlays were assessed in all 94 patients. The F-18 FDG uptake was graded (0, uptake in vessel wall ≤ mediastinum; 1, < liver; 2, = liver; and 3, > liver), with grades 2 or 3 considered positive for LVV [11]. A 10-mm circular volume of interests (VOIs) to assess SUVmax was manually defined for the following eight arterial segments allowing for a whole-body assessment of the entire vasculature: ascending aorta, aortic arch, descending and abdominal aorta, and vertebral, carotid, subclavian, and femoral arteries (in total, 752 VOIs).

All ROIs were set over the arterial segments carefully excluding adjacent tissue not related to the vessel walls. In addition, circular VOIs with a diameter of 2 cm were placed over bone marrow of lumbar vertebrae 5, the spleen, and the liver [12]. For all VOIs, mean standardized uptake values (SUVmean) were obtained. The vessel wall-to-liver ratio (VLR) of the SUV was calculated by dividing the vessel SUVmean by the liver, serving as a reference standard for the VLR calculation. Each VOI was placed in a consensus read, and the observers were blinded at the time of analysis. 

### 2.3. Statistical Analysis

The data were analyzed using Prism, version 8.4.2 (GraphPad, San Diego, CA, USA). Continuous variables are presented as the mean ± standard deviation. The VLRs of the vessel segments between the different treatment groups were compared using the Mann–Whitney *U*-test. Bonferroni adjustment was performed. Spearman’s correlation was used to determine the association between parameters. For comparison of three or more groups, one-way or two-way ANOVA with Bonferroni’s post hoc was used. A *p*-value of less than 0.05 was assumed to be statistically significant.

## 3. Results

### 3.1. Impact of Immunomodulatory Drugs on Vessel Wall Uptake

At the time of PET/CT, 40/94 (42.6%) patients did not receive any treatment, while the remaining 54/94 (57.4%) patients were taking immunomodulatory drugs according to current guidelines (including prednisone, methotrexate, or other conventional immunosuppressive agents) [4]. The patients were then grouped according to type of medication as follows: Group 1 taking prednisone daily (29/54 (53.7%)); Group 2 with daily prednisone and methotrexate intake (once per week, 9/54 (16.7%)); and Group 3 receiving daily prednisone plus other immunomodulatory drugs (11/54 (20.4%)), including the daily intake of mycophenolate mofetil, azathioprine, leflunomide, and rituximab every second week).

In Group 4 (5/54 (9.3%)), none of the patients received GC, instead they received methotrexate or cyclophosphamide alone, and this group was excluded from further analyses due to the low number of subjects assigned to this group (Figure 1). The daily GC doses among all subcohorts were as follows: In Group 1, 20 ± 7.1 mg; in Group 2, 8.9 ± 3.9 mg; and in Group 3, 10.4 ± 7.1 mg. The treatment duration could not be established in all subjects given the retrospective nature of the present study. The CRP (mg/L) as a functional systemic biomarker was also recorded in 87/94 (92.6%) at the time of PET/CT.

Table 2 provides an overview of the quantitative parameters derived from all vessel segments and hematopoietic organs of the entire cohort.

#### Vessel Wall Uptake Decreases with the Additional Intake of Immunomodulatory Drugs

**Figure 2:**
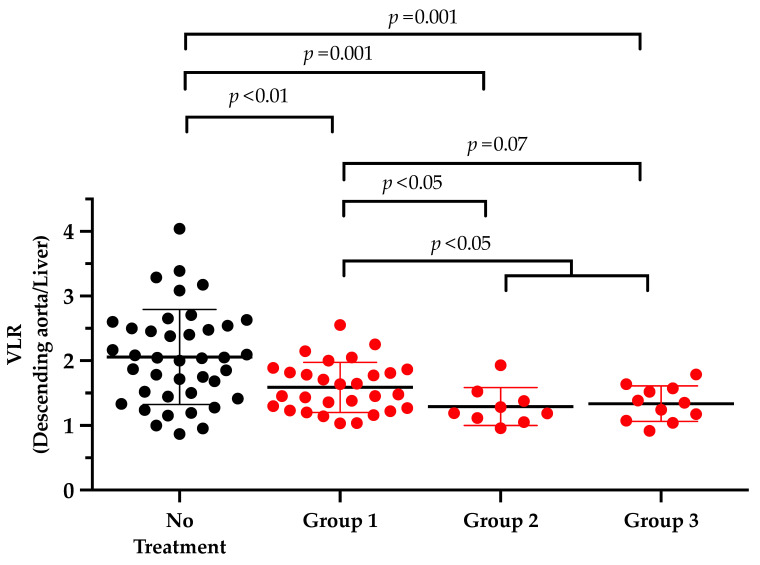
Quantitative assessment of the most profoundly affected vessel segment (descending aorta). Comparison of patients with no treatment vs. subjects receiving prednisone alone (Group 1), individuals with methotrexate intake in combination with prednisone (Group 2) or other immunomodulatory drugs (mycophenolate mofetil, azathioprine, rituximab, and leflunomide) in combination with prednisone (Group 3) at the time of PET/CT. Uptake in vessel walls decreased with the additional intake of immunomodulatory drugs.

Investigating treatment-naïve subjects, the mean VLR was almost consistently above 1 for all analyzed vessel walls (8/8 (100%), VLR among all segments, 1.81 ± 0.74). The VLR, however, substantially varied among patients, with the highest VLR in the descending (range, 0.87–4.04, Figure 2) and abdominal aorta (range, 0.77–4.19). In addition, patients with no immunomodulatory treatment at the time of PET/CT also demonstrated increased VLR in all arterial segments relative to patients under medication (Table 3). When compared to treatment-naïve patients, patients allocated to Group 1 demonstrated significantly lower differences in VLR in 5/8 (62.5%) arterial segments (*p* ≤ 0.02). For Group 2, 6/8 (75%) arterial segments also had significant lower VLR compared to untreated subjects (*p* ≤ 0.006), while for Group 3, 7/8 (87.5%) arterial segments were significantly lower relative to the treatment-naïve group (*p* ≤ 0.05; Table 3).

**Figure 3:**
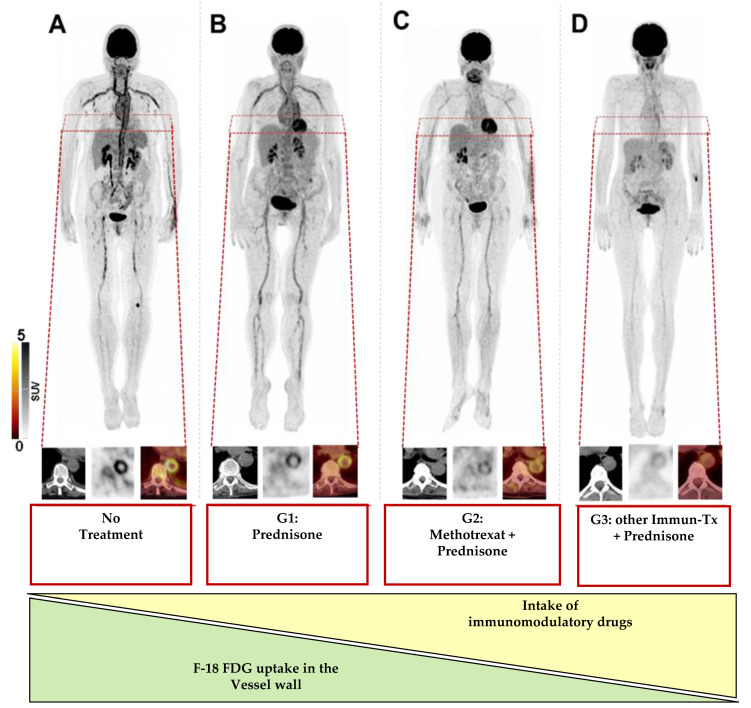
Maximum-intensity projections and magnified descending aorta in patients with (**A**) no treatment, (**B**) receiving prednisone alone (Group 1), (**C**) methotrexate in combination with prednisone (Group 2), or (**D**) prednisone in combination with other immunomodulatory drugs (Immuno-Tx, including mycophenolate mofetil, azathioprine, rituximab, and leflunomide) at the time of PET/CT (Group 3). The uptake in vessel walls decreased with the additional intake of immunomodulatory drugs. For every case, computed tomography (CT), positron emission tomography (PET), and PET/CT are displayed.

Among all vessels, one of the most prominent differences in VLR were achieved in the descending aorta for both the Group 1 and 3 (*p* ≤ 0.001), whereas in Group 2, the most significant decline was observed in the subclavian artery (*p* = 0.0005), followed by the descending aorta (*p* = 0.001, Figure 2). Additionally a comparison between Group 1 and a combination of Group 2 and 3 showed a significant correlation in 4/8 (50%) segments (*p* < 0.2, carotid artery, ascending artery, aortic arch, and descending artery). Figure 3 displays the maximum intensity projections of a treatment-naïve patient at the time of scan compared to the subjects allocated to Groups 1–3.

### 3.2. Hematopoietic Organs

#### Uptake in Vessel Wall Segments Correlated with Signals from Hematopoietic Organs

**Figure 4:**
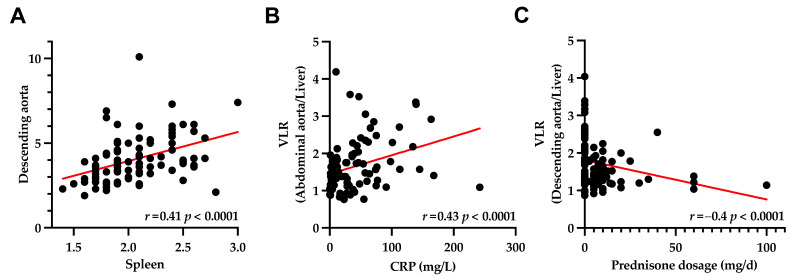
(**A**) Uptake in the descending aorta correlated significantly with the uptake in the spleen. (**B**) C-reactive protein as a marker of systemic inflammatory response correlated significantly with vessel wall-to-liver ratio (VLR) of abdominal aorta. (**C**) VLR of descending aorta correlated with the prednisone dosage.

The splenic uptake was positively correlated with VLR in 8/8 (100%) arterial segments (*r* ≥ 0.34, *p* ≤ 0.0007; Figure 4A), whereas the uptake in bone marrow still reached significance in 3/8 (37.5%) of the investigated vessel walls (*r* ≥ 0.2, *p* ≤ 0.05; Table 4), with 2/5 (40%) vessel walls demonstrating a trend toward significance (*p* = 0.05, respectively). 

### 3.3. Systemic Inflammatory Response

#### Vessel Wall Uptake Correlated with Markers of Systemic Inflammatory Response and Prednisone Dosage

The serum CRP correlated significantly with the VLR in 8/8 (100%) arterial segments (*r* ≥ 0.25, *p* ≤ 0.05; Table 5, Figure 4B). The prednisone dosage and VLR were inversely correlated in 6/8 (75%) segments (*r* ≥ −0.4, *p* ≤ 0.05), with the most prominent correlation achieved for the descending aorta and aortic arch (*r* = −0.4, *p* < 0.0001, respectively; Table 6, Figure 4C).

## 4. Discussion

Using inflammation-targeted molecular imaging in a cohort of LVV patients, the F-18 FDG uptake in vessel walls substantially varied among patients. Compared to treatment-naïve subjects, the additional intake of immunomodulatory drugs also led to a substantial reduction of uptake among all investigated vessel walls. As such, the herein presented results suggest an incremental value of a whole-body molecular imaging-derived biomarker read-out of the entire inflammatory activity in patients diagnosed with LVV, even under treatment at the time of the scan.

Treatment initiation with GC is strongly endorsed already in patients presenting with signs and symptoms suggestive of LVV, leaving clinicians with the dilemma of scheduling patients for imaging while already placed under immunosuppression [4]. In the present analysis, a substantial cohort of 54 guideline-compatible treated LVV patients was investigated [4] and grouped according to the intake of immunomodulation. Serving as reference, we enrolled a LVV cohort with no treatment at the time of scan, and, similar to previous reports comparing subjects with LVV to randomly chosen cancer patients [13,14], a substantial reduction of vessel wall uptake under GC was noted (Table 3).

In active LVV, high doses of GC should be initiated as soon as possible to establish a robust remission [4], whereas refractory or relapsing disease requires further modifications, such as GC-dose escalation or a switch to another immunomodulatory pharmacological intervention [4]. These adjunctive and potentially GC-sparing agents include, but are not limited to, conventional synthetic disease modifying anti-rheumatic drugs, such as methotrexate (investigated in Group 2), mycophenolate mofetil, leflunomide, or azathioprine (analyzed in Group 3) [4].

As such, we not only demonstrated a lower F-18 FDG uptake in the vasculature of patients exclusively treated with GC (Group 1) but also provide evidence that the vessel wall uptake was significantly modified by the additional prescription of GC-sparing agents, such as methotrexate or azathioprine (Figure 3). Of note, such escalating treatment regimens beyond GC intake most likely reflect the clinical reality in patients with LVV [4], and thus an increased awareness of such a potential interpretive bias may be crucial for a more accurate scan assessment. Second, the herein presented findings also strongly suggest that a biomarker of local tissue inflammation opens avenues for imaging-guided therapeutic strategies that may help in selecting the individuals most likely to respond to therapy. 

Glucose consumption revealed by F-18 FDG PET/CT in vessel wall foci cannot reliably distinguish between inflammation or on-going healing under medication [15]. However, in the present analysis, a significant correlation with sources of inflammatory cells (spleen and bone marrow, Table 4) and CRP (Table 5) was found, further supporting the notion that the measured uptake in vessel walls actually reflects the LVV-triggered immune reaction [16]. Future studies, however, may also investigate the arterial wall of LVV patients with the novel CXCR4-ligand Ga-68 Pentixafor, which is expressed on a broad range of leukocytes [17,18], thereby, providing a more specific surrogate marker in inflammatory vascular disease [19].

Setting >750 ROIs in eight major arterial segments, we almost exploited the full potential of PET imaging to assess the entire inflammatory disease extent. Previous reports investigating the vessel wall uptake in LVV relied on a visual analysis considering a scan positive if a tree-root like uptake pattern was noted on a maximum-intensity projection [9]. The herein conducted in-depth quantification of the vessel wall uptake, however, revealed that the descending aorta and aortic arch had the most profound reduction of F-18 FDG uptake among all three treatment groups (Figure 2, Table 3). Second, a substantial correlation with splenic uptake (Figure 4A) and the most profound inverse correlation with GC dosage (Table 6) was noted. Thus, although a quantitative whole-body assessment is desirable, the descending aorta or the aortic arch may serve as attractive surrogate markers of disease extent, which could be implemented in clinical routine or future clinical trials. 

This study has several limitations: First, the present study is retrospective and monocentric. Second, the derived findings should be re-evaluated in an even higher number of subjects. Last, the GC dosage did not correlate with small vessels, such as vertebral or femoral arteries or the carotid artery, indicating that the uptake in such small areas of interest is potentially biased by a partial volume effect. The duration of treatment, however, may also have an impact on the herein derived findings and should also be assessed in future prospective studies. 

In addition, CRP was not available in seven subjects, and the correlations of the VLR with hematopoietic organs, CRP, and prednisone dosage were rather weak (*r* < 0.5) and, thus, of questionable biological significance. Further correlations of the vessel wall uptake with clinical parameters, such as the body mass index, erythrocyte sedimentation rates, or hyperlipidemia, should also be addressed in future studies. Moreover, a CT read-out may further help to distinguish between LVV-caused vessel wall uptake and atherosclerosis [20].

The present study aimed to investigate the impact of immunomodulatory interventions in LVV, but future evaluations may also categorize respective cohorts into patients afflicted with Takayasu’s Arteritis vs. Giant Cell Arteritis. In addition, in-depth heterogeneity assessments could be also conducted, e.g., by the reanalysis of mathematically extracted radiomic feature metrics or dual tracer read-outs allowing head-to-head comparisons of each vessel wall [19,21].

## 5. Conclusions

In the present study, we enrolled a cohort of LVV patients who were imaged with F-18 FDG, and a lower uptake in the vessel walls was linked to a higher intake of the immunomodulatory drugs typically prescribed in the clinic. As such, the present work suggests a potential bias regarding scan interpretation in subjects under guideline-compatible immunomodulation at the time of the scan. Second, the derived findings also lay the foundation for future studies investigating precision medicine algorithms in LVV based on targeted molecular imaging or to identify high-risk subjects prone to later adverse events.

## Figures and Tables

**Table 1:** Patient characteristics. Percentages are given in brackets.

Variable	Total	Mean ± SD
Clinical parameters		
Female	72/94 (76.6)	-
Age		63 ± 13
>50 years	82 (87.2)	-
≤50 years	12 (12.8)	-
C-reactive protein (mg/L)	87/94 (92.6)	42.8 ± 46.5
Diabetes ^1^	6/87 (6.9)	-
Hypertension ^2^	44/88 (50.0)	-
Medication		
No treatment	40/94 (42.6)	-
Treatment	54/94 (57.4)	-
Group 1: Prednisone	29/54 (53.7)	-
Group 2: Methotrexate with prednisone	9/54 (16.7)	-
Group 3: Other immunomodulatory drugs ^3^ with prednisone	11/54 (20.4)	-
Group 4: Methotrexate or Cyclophosphamide ^4^	5/54 (9.3)	-

^1^ Available in 87/94 subjects, respectively; ^2^ 88/94 subjects, respectively; ^3^ mycophenolate mofetil, azathioprine, rituximab, and leflunomide; ^4^ excluded from further analysis due to the low number of subjects.

**Table 2:** Standardized uptake values of the bone marrow, spleen, and each investigated arterial segment (entire cohort). The vessel wall-to-liver ratios (VLRs) derived from all arterial segments. The means ± SD are given (*n* = 94).

	SUV	VLR
Hematopoietic organs		
Bone marrow	1.82 ± 0.48	-
Spleen	2.06 ± 0.33	-
Arterial segments		
Vertebral artery	2.79 ± 1.47	1.18 ± 0.63
Subclavian artery	3.41 ± 1.61	1.46 ± 0.74
Carotid artery	3.52 ± 1.40	1.50 ± 0.62
Ascending aorta	3.87 ± 1.24	1.65 ± 0.54
Aortic arch	3.99 ± 1.17	1.70 ± 0.52
Descending aorta	4.04 ± 1.39	1.73 ± 0.62
Abdominal aorta	3.92 ± 1.64	1.68 ± 0.75
Femoral artery	3.19 ± 1.57	1.35 ± 0.65

**Table 3:** The vessel wall-to-liver ratio (VLR, mean ± SD) for subjects without treatment and different groups receiving either prednisone alone (Group 1), methotrexate in combination with prednisone (Group 2) or other immunomodulatory drugs in combination with prednisone at the time of PET/CT (Group 3). The p-values were derived from comparison of different groups vs. treatment-naïve patients at time of scan. The number of significant vessel segments increased with the additional intake of immunomodulatory drugs.

Arterial Segment	No Treatment	Group 1 Prednisone		Group 2 Methotrexate + Prednisone		Group 3 Other Immunomodulatory Drugs ^1^ + Prednisone	
	VLR	VLR	*p*-Value	VLR	*p*-Value	VLR	*p*-Value
Vertebral artery	1.35 ± 0.72	1.18 ± 0.67	0.28	0.98 ± 0.35	0.06	0.91 ± 0.24	**0.006**
Subclavian artery	1.84 ± 0.83	1.33 ± 0.54	**0.007**	0.90 ± 0.30	**0.0005**	1.00 ± 0.53	**0.001**
Carotid artery	1.76 ± 0.73	1.47 ± 0.49	0.06	1.13 ± 0.38	**0.006**	1.14 ± 0.27	**0.004**
Ascending aorta	1.90 ± 0.62	1.51 ± 0.42	**0.004**	1.31 ± 0.22	**0.003**	1.51 ± 0.29	0.07
Aortic arch	1.96 ± 0.58	1.63 ± 0.41	**0.018**	1.32 ± 0.26	**0.001**	1.35 ± 0.23	**0.0003**
Descending aorta	2.06 ± 0.73	1.59 ± 0.39	**0.005**	1.29 ± 0.29	**0.001**	1.34 ± 0.27	**0.001**
Abdominal aorta	2.08 ± 0.92	1.48 ± 0.43	**0.006**	1.21 ± 0.32	**0.002**	1.33 ± 0.46	**0.007**
Femoral artery	1.56 ± 0.78	1.26 ± 0.54	0.12	1.27 ± 0.45	0.42	1.02 ± 0.34	**0.049**

^1^ Mycophenolate mofetil, azathioprine, rituximab, and leflunomide reached significance (*n* = 94) indicated in bold.

**Table 4:** Correlations between the standardized uptake value (SUV) of each arterial segment, spleen, and bone marrow (*n* = 94).

Arterial Segment	Spleen		Bone Marrow	
	*r*	*p*-Value	*r*	*p*-Value
Vertebral artery	0.41	**<0.0001**	0.18	0.08
Subclavian artery	0.34	**0.0007**	0.12	0.27
Carotid artery	0.54	**<0.0001**	0.25	**0.014**
Ascending aorta	0.41	**<0.0001**	0.26	**0.0013**
Aortic arch	0.48	**<0.0001**	0.20	0.05
Descending aorta	0.41	**<0.0001**	0.08	0.44
Abdominal aorta	0.38	**0.0002**	0.22	**0.031**
Femoral artery	0.47	**<0.0001**	0.20	0.05

The reached significance is indicated in bold.

**Table 5:** Correlations between the vessel wall-to-liver ratio of each arterial segment with C-reactive protein (*n* = 87/94).

Arterial Segment	*r*	*p*-Value
Vertebral artery	0.32	**0.003**
Subclavian artery	0.33	**0.002**
Carotid artery	0.38	**0.0003**
Ascending aorta	0.25	**0.018**
Aortic arch	0.38	**0.0004**
Descending aorta	0.29	**0.007**
Abdominal aorta	0.43	**<0.001**
Femoral artery	0.35	**0.0008**

The reached significance is indicated in bold.

**Table 6:** Correlations between the vessel wall-to-liver ratio of each arterial segment with prednisone dosage (*n* = 94).

Arterial Segment	*r*	*p*-Value
Vertebral artery	−0.15	0.15
Subclavian artery	−0.38	**0.0002**
Carotid artery	−0.24	**0.02**
Ascending aorta	−0.26	**0.01**
Aortic arch	−0.4	**<0.0001**
Descending aorta	−0.4	**<0.0001**
Abdominal aorta	−0.36	**0.0004**
Femoral artery	−0.15	0.16

The reached significance is indicated in bold.

## Data Availability

The data are not publicly available as, due to the European regulations regarding data protection, we cannot make the data available online or send them. However, all data are available for revision on-site.
